# Ten years gone: Response to Cavve (2024)

**DOI:** 10.1177/10398562251322011

**Published:** 2025-02-18

**Authors:** Andrew James Amos

**Affiliations:** Townsville, QLD

Dear Editor,

I thank Cavve^1^ for his interest in my article reporting rapid increases in the patients, staff, and interventions across Australia’s public paediatric gender services.^2^ While his main criticism is that I grouped several patient subgroups under ‘clients’, Cavve doesn’t illustrate how this would have changed my results. Far from his assertion that ‘[Amos’s] conclusions…are not limited to data availability or quality but instead make specific statements on trends as if the data were accurate and reliable’,^1^ my abstract explicitly draws attention to the fact that ‘[l]imited transparency makes it difficult to judge the number of patients seen, treatments provided, and outcomes achieved [by gender services]’.^2^

Although Cavve^1^ makes the strong claim that my article failed to honour ‘an ethical responsibility to ensure [its] reporting…meets data integrity and accuracy standards’, he rather undermines the charge by acknowledging that his own analysis was based on my provision to him of all the data reported, alongside the FoI requests that produced them. During our correspondence, I provided all my spreadsheets with workings, outlined simplifying assumptions I had made, and provided their justifications. Short of directly overseeing his analysis, I’m not sure what else I could have done to assist Cavve in reaching an accurate understanding of their integrity, but he provides no examples of failure to meet data integrity or accuracy standards in my work.

Perhaps inadvertently, Cavve’s^1^ Table 1 directly illustrates the key points of my article and provides a compelling rationale for reporting a single group of ‘clients’ rather than separate subgroups. It proves that the best information we have about the number and type of patients seen by public paediatric gender clinics, as well as what is done to those patients, is completely inadequate. Nevertheless, in the absence of any other evidence, it is justifiable to rely upon the FoI data as most reliable indication of the number of paediatric patients being seen by gender clinics in WA as elsewhere in Australia.

It is interesting that Cavve^1^ suggests that some sort of collaboration to establish a robust paediatric gender medicine dataset has recently been funded, albeit the reference supplied was not retrievable. As there have been multiple examples of gender-affirming care advocates suppressing research when it doesn’t support increasing gender-affirming care,^3–5^ it seems prudent to await the results of this collaboration before assuming it will solve the problems identified in my article.

Since my article was published, the authoritative Cass Review into paediatric gender medicine has shown the model of care for all Australia’s public paediatric gender clinics^6^ have extremely low methodological quality and editorial independence.^4^ In addition, Cass pointed out that the gender-affirming care model used in Australia’s clinics had expanded far more rapidly than other treatments despite a complete absence of strong evidence that they improve the health or mental health of gender dysphoric minors.

Thus, contra Cavve,^1^ Cass and numerous other international reviews show it is evidence-based medicine, not activism, that demands the restriction of gender-affirming care.^4^
